# Auditory Tests for Characterizing Hearing Deficits in Listeners With Various Hearing Abilities: The BEAR Test Battery

**DOI:** 10.3389/fnins.2021.724007

**Published:** 2021-09-29

**Authors:** Raul Sanchez-Lopez, Silje Grini Nielsen, Mouhamad El-Haj-Ali, Federica Bianchi, Michal Fereczkowski, Oscar M. Cañete, Mengfan Wu, Tobias Neher, Torsten Dau, Sébastien Santurette

**Affiliations:** ^1^Hearing Systems Section, Department of Health Technology, Technical University of Denmark, Kgs. Lyngby, Denmark; ^2^Interacoustics Research Unit, Kgs. Lyngby, Denmark; ^3^Institute of Clinical Research, University of Southern Denmark, Odense, Denmark; ^4^Oticon Medical, Smørum, Denmark; ^5^Research Unit for ORL-Head & Neck Surgery and Audiology, Odense University Hospital & University of Southern Denmark, Odense, Denmark; ^6^Centre for Applied Audiology Research, Oticon A/S, Smørum, Denmark

**Keywords:** audiology, hearing loss, loudness, binaural processing, speech perception, spectro-temporal resolution, auditory profile

## Abstract

The Better hEAring Rehabilitation (BEAR) project aims to provide a new clinical profiling tool—a test battery—for hearing loss characterization. Although the loss of sensitivity can be efficiently measured using pure-tone audiometry, the assessment of supra-threshold hearing deficits remains a challenge. In contrast to the classical “attenuation-distortion” model, the proposed BEAR approach is based on the hypothesis that the hearing abilities of a given listener can be characterized along two dimensions, reflecting independent types of perceptual deficits (distortions). A data-driven approach provided evidence for the existence of different auditory profiles with different degrees of distortions. Ten tests were included in a test battery, based on their clinical feasibility, time efficiency, and related evidence from the literature. The tests were divided into six categories: audibility, speech perception, binaural processing abilities, loudness perception, spectro-temporal modulation sensitivity, and spectro-temporal resolution. Seventy-five listeners with symmetric, mild-to-severe sensorineural hearing loss were selected from a clinical population. The analysis of the results showed interrelations among outcomes related to high-frequency processing and outcome measures related to low-frequency processing abilities. The results showed the ability of the tests to reveal differences among individuals and their potential use in clinical settings.

## 1. Introduction

In current clinical practice, hearing loss is diagnosed mainly on the basis of pure-tone audiometry (ISO 8253-1, 2010). The audiogram helps differentiate between conductive and sensorineural hearing losses and can characterize the severity of the hearing loss from mild to profound. However, the pure-tone audiogram only assesses the sensitivity to simple sounds, which is not necessarily related to listening abilities at supra-threshold sound pressure levels (e.g., a person's ability to discriminate speech in noise).

Pure-tone audiometry is often complemented by speech audiometry (ISO 8253-3, 2012), which is a test typically performed in the form of word recognition performance in quiet (Anderson et al., 2018). Although this test can provide information about supra-threshold deficits (Gelfand, 2009), measurements of speech understanding in noise have been found more informative (Nilsson et al., 1994; Killion et al., 2004). Since improving speech intelligibility is usually the main goal of successful hearing rehabilitation, several auditory factors affecting speech intelligibility in noise have been investigated (e.g., Glasberg and Moore, 1989; Houtgast and Festen, 2008; Strelcyk and Dau, 2009). Audibility (in conditions with fluctuating maskers), frequency selectivity (in conditions with stationary noise), and temporal processing acuity (in conditions with speech interferers) have been identified as important factors affecting speech reception thresholds in noise when using meaningful sentences as speech material (e.g., Rhebergen et al., 2006; Oxenham and Simonson, 2009; Johannesen et al., 2016; Desloge et al., 2017)^1^. Thus, a hearing evaluation that goes beyond pure-tone sensitivity and speech intelligibility in quiet would be expected to provide a more accurate characterization of a listener's hearing deficits.

In Denmark, the Better hEAring Rehabilitation (BEAR) project was initiated with the aim of developing new diagnostic tests and hearing-aid compensation strategies for audiological practice. Although the assessment of individual hearing deficits can be complex, new evidence suggests that the perceptual consequences of a hearing loss can be characterized effectively by two types of hearing deficits, defined as “auditory distortions” (Sanchez-Lopez et al., 2018). By analyzing the outcomes of two previous studies (Johannesen et al., 2016; Thorup et al., 2016) with a data-driven approach, Sanchez-Lopez et al. (2018) identified high-frequency (HF) hearing loss as the main predictor of one of the distortions, whereas the definition of the second type of distortion was inconclusive. The inconclusiveness in the prediction of the second distortion was most likely due to differences between the two studies in terms of hearing loss profiles and outcome measures. Here, a new dataset was therefore collected based on a heterogeneous group of listeners with audiometric hearing losses ranging from very mild to severe and with a large range of audiometric profiles. To that end, the most informative tests resulting from the analysis of Sanchez-Lopez et al. (2018) were included, together with additional auditory tests that had shown potential for hearing profiling in other previous studies. The tests included in the current study are referred to as the BEAR test battery.

The characterization of hearing deficits beyond the audiogram was considered in several earlier studies (e.g., Saunders et al., 1992; Santurette and Dau, 2012; Lecluyse et al., 2013; Brungart et al., 2014; Esch and Dreschler, 2015; Rönnberg et al., 2016). Among them, the HEARCOM project (Vlaming et al., 2011) proposed an extended hearing profile formed by the results of several behavioral tests. These tests targeted various auditory domains, such as audibility, loudness perception, speech perception, binaural processing, and spectro-temporal resolution, as well as a test of cognitive abilities. Importantly, while the auditory domains considered in the BEAR test battery are similar to the ones considered in the HEARCOM project, the BEAR project aims to additionally classify the patients in subcategories and to create a link between hearing capacities and hearing-aid parameter settings.

The tests included in the BEAR test battery were chosen based on the following criteria: (1) There is evidence from the hearing research literature that the considered test is informative (i.e., it provides information about the individual hearing deficits) and reliable (i.e., the result of the test does not vary over time). (2) The outcomes of the test may be linked to a hearing-aid fitting strategy. (3) The outcome measures are easy to interpret and to explain to the patient. (4) The task is reasonably time-efficient or can be suitably modified to meet this requirement (e.g., by changing the test paradigm or developing an out-of-clinic solution). (5) The test implementation can be done with equipment available in clinics. (6) The tasks are not too demanding for patients and clinicians. (7) Tests with several outcome measures are prioritized. (8) The language-independent tests are also prioritized. Although a large list of tests candidates was considered in an early stage, discussions among the authors and other BEAR partners to shorten the list led to the current proposal. Some classical tests were discarded because a suitable alternative was more promising. For example, the short-increment sensitivity index (SISI test) was discarded since there was a more informative candidate for measuring loudness perception.

The selected test battery included measures of audibility, loudness perception, speech perception, binaural processing abilities, spectro-temporal modulation (STM) sensitivity, and spectro-temporal resolution. It was implemented and tested in older listeners with different hearing abilities (from mild to severe hearing losses) as a representative sample of the population of hearing-aid user candidates mainly affected by age-related hearing loss. The goals of the study were as follows: (1) To collect reference data from a representative sample of HI listeners for each of the selected tests, (2) to analyze the test–retest reliability of these tests, (3) to analyze the relationships between the different outcome measures, and (4) to propose a version of the test battery that can be implemented in hearing clinics.

## 2. Overview of the Test Battery

The test battery consisted of ten tests (9 tests besides the pure-tone audiometry). The outcomes of the proposed tests are divided into six categories. Table 1 shows the tests and the corresponding auditory domains or categories. For convenience, the domains spectro-temporal modulation sensitivity and spectro-temporal resolution are presented together in the category spectro-temporal processing. The following sections introduce the experimental methods and present all tests individually. The dataset is publicly available in a Zenodo repository (Sanchez-Lopez et al., 2021b). More details about the method can be found in the Supplementary Material in the data repository.

**Table 1:** List of the tests included in the BEAR test battery and their corresponding auditory domains.

**Test name**	**Category**	**Variables**
Pure-tone audiometry^a^	Audibility	AUD_LF_, AUD_HF_
Extended audiometry at high frequencies ^b^		FLFT
Adaptive categorical		HTL_LF_, HTL_HF_
loudness scaling^c^	Loudness	MCL_LF_, MCL_HF_
	perception	DynR_LF_, DynR_HF_
		Slope_LF_, Slope_HF_
Word recognition scores^d^	Speech	SRT_Q_, maxDS
Hearing in noise test^e^	Perception	SRT_N_, SScore^+4dB^
Spectro-temporal modulation test^i^		sSTM_8_, sSTM_4k_
	Spectro-	fSTM_8_, fSTM_4k_
Extended audiometry in noise ^j^^,^^k^^,^^l^	temporal	TiN_LF_,TiN_HF_
	processing	SMR_LF_, SMR_HF_,
		TMR_LF_, TMR_HF_
Maximum frequency for IPD detection^f^	Binaural	IPD_fmax_
Binaural pitch^g^	processing	BP_20_
Extended binaural audiometry in noise^h^	abilities	BMR

*LF, Lower-frequencies, HF, higher-frequencies*.

*AUDx, Pure-tone average at low (x=LF; f ≤ 1kHz) or high (x = HF; f > 1kHz) frequencies. // FLFT: Fixed-level frequency threshold at 80 dB SPL. ACALOS outcome variabless are averaged for low (x=LF; f ≤ 1kHz) and high (x = HF; f > 1kHz) frequencies. // HTL, Hearing threshold level MCL: Most comfortable level / Slope: Slope of the loudness function / DynR: Dynamic range // SRT_Q_: Speech reception threshold in quiet / maxDS: Maximum speech discrimination score. // SRT_N_: Speech reception threshold in noise / SScore^+4dB^: Sentence recognition score at +4 dB SNR // sSTM: Sensitivity for detecting a spectro-temporally modulated noise at 20log(m) = -3 dB, where m is the modulation depth / fSTM: Fast version of the STM test (Bernstein et al., 2016) // Extended audiometry outcome measures were measured at 0.5 kHz (x=LF) and at 2 kHz (x=HF) // eAUD-N: Tone detection in TEN noise // TMR: Temporal masking release // SMR: Spectral masking release // IPD_fmax_: Frequency threshold for detecting an interaural phase difference of 180°. // BP_20_: Binaural pitch detection scores for 20 presentations // BMR: Binaural masking release*.

a *ISO 8253-1, 2010*;

b *Rieke et al., 2017*;

c *Brand and Hohmann, 2002*;

d *ISO 8253-3, 2012*;

e *Nielsen and Dau, 2011*;

f *Füllgrabe and Moore, 2017*;

g *Santurette and Dau, 2012*;

h *Durlach, 1963*;

i *Bernstein et al., 2016*;

j *Moore et al., 2000*;

k *Schorn and Zwicker, 1990*;

l *van Esch and Dreschler, 2011*.

### 2.1. Reference Data From Younger Normal-Hearing Listeners

Although many of the tests included in the test battery are based on previous studies with normative data, a group of 11 young normal-hearing (NH) listeners were tested in the facilities of the Technical University of Denmark (DTU) and the University of Southern Denmark (SDU) to obtain reference data for this specific implementation of each of the tests. The summary statistics of the outcome variables from Table 1 for these NH listeners are shown in Table 2.

**Table 2:** Reference data of the young normal-hearing group.

**Outcome**	**5th**	**25th**	**50th**	**75th**	**95th**	**Unit**
AUD_LF_	–5	0	0	5	10	dB HL
AUD_HF_	–10	0	5	10	10	dB HL
FLFT	14.42	15.60	17.88	18.02	19.31	kHz
HTL_LF_	–7.5	–2.5	1.25	2.5	7.5	dB HL
HTL_HF_	–5.5	2.5	8.75	15	23.5	dB HL
MCL_LF_	55	62.5	70	72.5	78	dB HL
MCL_HF_	62.5	70	75	80	87.5	dB HL
DynR_LF_	87.5	92.5	97.5	105	117.5	dB
DynR_HF_	74.5	85	92.5	100	105	dB
SLope_LF_	0.27	0.30	0.33	0.37	0.42	CU/dB
Slope_HF_	0.27	0.30	0.33	0.37	0.44	CU/dB
SRT_Q_	6	10	14	18	21	dB SPL
maxDS	96.7	100	100	100	100	% Corr
SRT_N_	–3.48	–1.84	–0.85	–0.19	1	dB SNR
SScore^+4dB^	80	90	95	100	100	% Corr
sSTM_8_	2.40	3.07	3.07	3.07	3.07	d'
sSTM_4k_	0.30	2.40	3.07	3.07	3.07	d'
fSTM_8_	–13.5	–12.7	–12.3	–10.5	–6.1	dB ML
fSTM_4k_	–9.1	–6.5	–4.375	–3.375	–2.7	dB ML
TiN_LF_	64.25	66.5	68.25	69.5	73.6	dB HL
TiN_HF_	64.1	68	69.375	70	73.85	dB HL
TMR_LF_	3.55	7.4167	9.375	13	15.95	dB
TMR_HF_	3.1	8.25	10.875	13	18.15	dB
SMR_LF_	16.55	19.75	21.75	23.25	26.15	dB
SMR_HF_	19.6	26	28.5833	30.75	31.25	dB
IPD	0.86	1.20	1.22	1.29	1.40	kHz
BP_20_	50	98.75	100	100	100	% Corr
BMR	14.3	15.875	17	18.4375	22.1	dB

*The data are shown as the 5th, 25th, 50th, 75th, and 95th percentiles. In tests performed monoaurally, the summary corresponds to the data of both ears merged together (i.e., 22 ears)*.

### 2.2. Time Efficiency of the Test Battery

The examiners kept track of the time used by each of the participants in completing the test battery. In the case of unexpected events (e.g., unexpected or incongruent results), these events were cautiously annotated for later investigation. Regarding the test procedure, additional repetitions of the threshold estimations were needed if: (1) a repetition was considered as an outlier (i.e., a given threshold was greater than three scaled median absolute deviations of the two repetitions); or (2) the responses of the listeners during the tracking procedure were inconsistent or reached the maximum or minimum possible values. In that case, the measurement was considered an invalid or “missing” data point.

**Figure 1:**
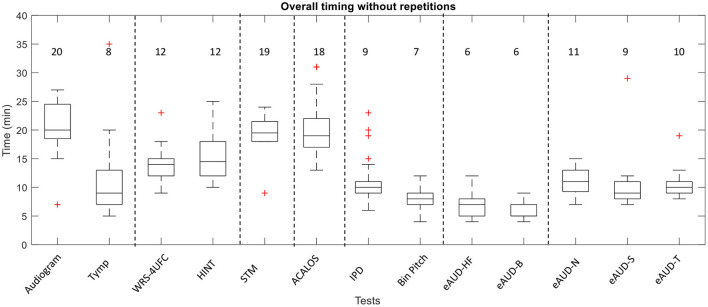
The overall time of the different tests in the test battery including the instructions. The data correspond to the annotations of the examiners. The basic examination with the audiometry and the tympanometry (Tymp) are included. The numbers represent the rounded median in minutes.

The timing of the individual tests is shown in Figure 1. Besides, the probability of needing an additional measurement and mean number of extra repetitions per listener are shown in Table 3. The repetitions were only suggested when the test was done using the alternative-forced choice (AFC) framework (i.e., the IPD test, the STM test, and the eAUD test in all the conditions). The total testing time was approximately 2.5 h excluding the initial interview, information about the study, and preparations.

**Table 3:** Table with the probability of needing repetitions (PR), and the probability of having missing values (PM).

**Test**	**PR (%)**	**PM (%)**	**PT (%)**	**E.Rep**.
STM	42.86	90.79	88.16	4.32
IPD	10.77	10.97	20.55	1.87
eAUD-HF	5.63	4.05	9.46	1.85
eAUD-N	66.67	46.58	82.19	3.00
eAUD-S	48.57	52.70	75.68	3.07
eAUD-T	53.85	46.58	75.34	3.27
S_0_N_0_	42.59	27.03	58.11	2.00
S_π_N_0_	20.59	9.11	27.03	1.85

*The total probability of repetitions (PT). The mean number of extra repetitions (E. Rep)*.

## 3. General methods

### 3.1. Participants and General Setup

**Figure 2:**
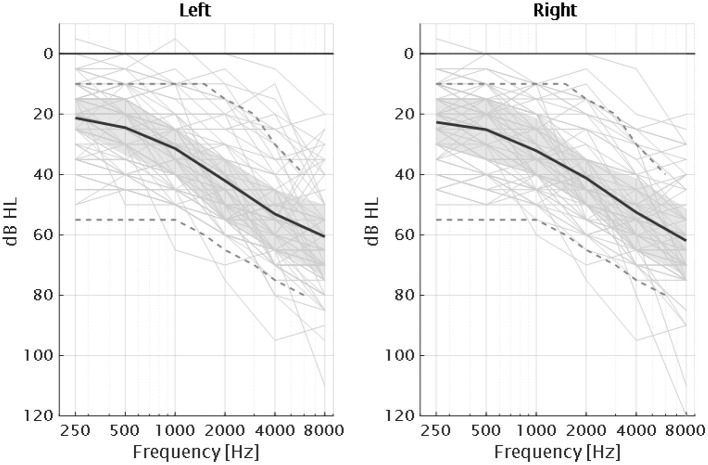
Audiograms of the 75 participants of the study together with the average for each ear (dark solid lines) and interquartile ranges (gray areas). The gray dashed lines correspond to the standard audiograms N1 and N4 according to the IEC60118-15 (Bisgaard et al., 2010).

Seventy-five listeners (38 of them females) participated in the study, who were aged between 59 and 82 years (median: 71 years). Five participants were considered older normal hearing (ONH) with thresholds below 25 dB hearing level (HL) in the frequency range between 0.25 and 4 kHz in both ears and no larger than 40 dB HL at 8 kHz (PTA ≤ 22 dB HL)^2^^2^. PTA was defined as the pure-tone average between 0.5, 1, and 2 kHz as is typically reported (Vermiglio et al., 2020). Two of these participants were not usual hearing-aid users. The hearing-impaired listeners (HI) group consisted of 70 participants with symmetric sensorineural hearing losses. Symmetric sensorineural hearing loss was defined as an interaural difference (ID) ≤ 15 dB HL at frequencies below 8 kHz and ID ≤ 25 dB HL at 8 kHz and air-bone gap ≤ 10 dB HL. The pure-tone audiograms of the participants are shown in Figure 2. The participants eligible for the present study had audiometric thresholds ≤ 55 dB HL (pure-tone audiometry not older than 1 year) in the range between 125 and 1,000 Hz. Participants with a pure tone threshold ≥ 75 dB HL at 2 kHz were excluded from the study as it was unlikely that it would be feasible to perform all the tests due to audibility issues.

The participants were recruited from the BEAR database (Houmoller et al., 2021) at Odense University Hospital (OUH), from the patient database at Bispebjerg Hospital (BBH), and from the database at the Hearing Systems Section at the Technical University of Denmark (DTU). The study was approved by the Science-Ethics Committee for the Capital Region of Denmark, H-16036391. All participants gave written informed consent and some of them received economical compensation for their participation, depending on each test site's regulations and whether the participant chose to participate without compensation.

### 3.2. Equipment

The basic audiological assessment consisted of pure-tone audiometry, wideband tympanometry (Rosowski et al., 2013) and middle ear muscle reflex, and was conducted in the facilities of OUH, BBH, and DTU. The rest of the tests were performed via PC in a double-walled sound-insulated booth (BBH and DTU) or in a small anechoic chamber (OUH). The tests were implemented in Matlab with a graphical user interface (GUI) that the examiner could operate without programming experience. Most of the tests were implemented using a modular framework for psychoacoustic experiments (AFC; Ewert, 2013), except for HINT, provided by Jens Bo Nielsen and Binaural Pitch test which was a reimplementation of the Binaural Pitch Test v1.0, Bispebjerg hospital, 2008. The participants were seated in the room and the stimuli were presented through headphones (Sennheiser HDA200) connected to a headphone-amplifier (SPL phonic) and an audio interface (RME Surface 24-bit). The equipment was calibrated using an artificial ear according to IEC 60318-1:2009. The tests consisting of threshold estimation using the AFC framework were repeated at least two times and the mean of the two measurements was considered as the final value. To ensure the quality of the data collected, additional repetitions were suggested by the framework until a certain standard deviation across measures was achieved.

### 3.3. Analysis of Test Reliability

The test–retest reliability of the test battery was assessed using intraclass correlation coefficients (ICC; Koo and Li, 2016), and the standard error of measurement (SEM; Stratford and Goldsmith, 1997). Since the ICC can be prone to misleading results in the case of systematic biases, the differences in the means were evaluated as well. The only tests with a likely bias were the individual measures of the extended audiometry at lower frequencies. More details can be found in Supplementary Table 1. It was of special interest to test the reliability in older listeners with different hearing abilities. Therefore, test–retest measurements were performed with a subgroup consisting of 11 participants for all tests of the test battery (excluding the screening spectro-temporal modulation test). According to the hearing thresholds estimated with ACALOS, two of those listeners had near-normal hearing thresholds at all tested frequencies, three had average hearing thresholds > 30 dB HL, and the remaining 6 listeners presented gently sloping hearing thresholds < 55 dB HL at all frequencies. The participants were aged between 59 and 82 years (median 69 years). The retest session was conducted within 4 months after the first visit.

## 4. High-Frequency Audibility

Recently, elevated thresholds at HFs (> 8 kHz) have been linked to the concept of “hidden hearing loss” and synaptopathy (Liberman et al., 2016). However, the measurement of audiometric thresholds above 8 kHz is not part of the current clinical practice. The fixed-level frequency threshold (FLFT) has been proposed as a quick and efficient alternative to HF audiometry (Rieke et al., 2017; Prendergast et al., 2020). The test is based on the detection of a tone presented at a fixed level. The frequency of the tone is varied toward HFs and the maximum audible frequency at the given level is estimated in an adaptive procedure. Here, a modified version of FLFT, using warble tones presented at 80 dB SPL, was used as the extended audiometry at high frequencies (eAUD-HF).

### 4.1. Method

The procedure used here was a yes/no task using a single-interval adjustment matrix (SIAM) yes-no task procedure (Kaernbach, 1990). As in traditional up-down procedures, the target can be presented in a given trial or not. If the target was detected, the frequency of the warble tone was increased according to a given step size; if it was not detected, the frequency was decreased. However, the adjustment of the target depends on the participant's behavior across trials, characterized by the false alarm rate (catch trials) and the hit rate. If the participant gives unbiased responses and keeps the criterion, the tests is an adaptive procedure similar to a 1-up 1-down. When the participant is caught, the step size becomes double. For each run, the first four reversals were discarded, and the threshold of each trial was calculated as the average of the two subsequent reversals. In the catch trials, no sound was presented. The warble tone *wt*(*f*_*c*_, *t*) was defined by the expression 1:


(1) $$\begin{array}{l} wt\left(f_{c},t\right)=\sin\left(2\pi f_{c}t+\frac{f_{c}f_{e}}{f_{r}}\sin\left(2\pi f_{r}t\right)\right), \end{array}$$

where *f*_*c*_ is the stimulus frequency, *f*_*r*_ = 4*Hz* is the frequency rate, and *f*_*e*_ = 4.3% is the frequency excursion of the frequency modulation.

### 4.2. Results and Discussion

The results of the FLFT measured at 80 dB SPL are shown in Table 4.

**Table 4:** Summary of the results of the extended audiometry for high frequencies eAUD-HF.

			**ONH**			**HI**		
**Outcome**	**Level**	**Ear**	**Mean (SD)**	**Q1**	**Q3**	**Mean (SD)**	**Q1**	**Q3**
**measure**								
eAUD-HF	80	LE	10.9 (1.2)	10.2	11.9	7.57 (2.7)	5.3	10
FLFT (kHz)	dB SPL	RE	11.7 (1.1)	10.9	12.5	8.12 (2.3)	6.7	10.2

*The results are shown as the mean, standard deviation, first and third quantiles (25 and 75th percentiles) for each ear*.

The maximum frequency threshold for a tone presented at 80 dB SPL (eAUD-HF) was 11 kHz for the ONH listeners and 8 kHz for the HI listeners. The HI group showed larger variability compared to the ONH group (interquartile range: 6 vs. 10 kHz). The eAUD-HF test showed very good reliability (ICC = 0.89; SEM = 495 Hz). These results suggest that the FLFT paradigm might be a good time-efficient alternative to the traditional audiometry for measuring HF sensitivity. A recent study pointed out the importance of off-frequency listening and the role of the excitation of the basal cochlea when presenting narrow-band stimuli in high levels (Encina-Llamas et al., 2019). Knowing the hearing sensitivity at HFs of a given patient might be crucial for better understanding of their supra-threshold deficits. Moreover, the eAUD-HF can include different levels and be useful not only for ototoxicity monitoring but also in association with other supra-threshold measures. For example, if FLFT is measured at a conversational level (i.e., 65 dB SPL), or at frequency-dependant levels corresponding to the speech spectrum, this measure could help to estimate the contribution of audible off-frequency listening to speech intelligibility or loudness perception.

## 5. Loudness Perception

Loudness perception can substantially differ between NH and HI listeners and has been connected to the peripheral non-linearity (e.g., Jürgens et al., 2011). While the growth of loudness shows a non-linear behavior in a healthy ear, the results from HI listeners suggest that loudness perception becomes linear when outer-hair cell (OHC) function is affected (e.g., Moore, 2007). Besides, the possibilities of characterizing hearing deficits, the loudness function can be used for fitting hearing aids (e.g., Oetting et al., 2018). Adaptive categorical loudness scaling (ACALOS; Brand and Hohmann, 2002) is the reference method for the current standard (ISO 16832, 2006) for loudness measurements.

### 5.1. Methods

According to the ACALOS method, 1/3-octave band noise was presented sequentially and the participant had to judge the perceived loudness using a 11-category scale ranging from “not heard” to “extremely loud.” The presentation level of the next stimulus was calculated based on the previous trials. The raw results, which correspond to categorical units (CU) spanned between 0 and 50, were fitted to a model of loudness as described in Oetting et al. (2014). The outcome measures of the ACALOS presented here are the most comfortable level (MCL), the slope of the loudness function (Slope), and the dynamic range (DynR) defined as the difference between the uncomfortable level (50 CU) and the hearing threshold (0.5 CU). Low-frequency (LF) average corresponds to the stimuli centered at 250, 500, and 1,000 Hz, and HF average corresponds to the stimuli centered at 2, 4, and 6 mkHz.

### 5.2. Results and Discussion

The results of the ACALOS outcome measures are shown in Table 5. The hearing thresholds (HTL) estimated by ACALOS were significantly correlated with the pure-tone audiometric thresholds (*ρ* = 0.88;*p* < < 0.0001) even when looking at the HI group alone (*ρ* = 0.83;*p* < < 0.0001) despite the use of different stimuli and procedure.

**Table 5:** Summary of the results of the adaptive categorial loudness scaling test (ACALOS).

			**ONH**			**HI**		
**Outcome**	**Freq**.	**Ear**	**Mean (SD)**	**Q1**	**Q3**	**Mean (SD)**	**Q1**	**Q3**
**measure**	**Range**							
MCL (dB HL)	LF	LE	81.5 (14.8)	73.3	84.1	80.6 (8.4)	76.4	85.8
		RE	76.5 (13.2)	70	80	79.1 (7.9)	74.7	84.1
	HF	LE	79.0 (17.6)	66.6	90.8	82.7 (12.3)	75.8	90
		RE	73.8 (17.2)	65	80	80.3 (9.9)	74.7	87.5
Slope (CU/dB)	LF	LE	0.35 (0.1)	0.3	0.4	0.45 (0.1)	0.3	0.5
		RE	0.36 (0.1)	0.3	0.4	0.48 (0.2)	0.3	0.5
	HF	LE	0.45 (0.1)	0.3	0.4	0.84 (0.5)	0.5	0.9
		RE	*0.41 (0.1)*	0.3	0.4	0.81 (0.4)	0.5	0.9
DynR (dB HL)	LF	LE	91.5 (16.8)	78.3	97.5	*76.7 (15.8)*	*64.5*	88.3
		RE	91.1 (18.8)	79.1	100	73.9 (16.0)	61.6	86.8
	HF	LE	77.6 (18.2)	72.5	85.8	50.8 (15.1)	*40.6*	60.2
		RE	78.6 (17.9)	67.5	*90.8*	50.7 (15.5)	38.9	60.4

*The results of the most comfortable level (MCL), slope of the growth of loudness (Slope), and dynamic range (DynR) are shown for low frequency (LF) and high frequency (HF) as the mean, standard deviation, first, and third quantiles (25 and 75th percentiles) for each ear*.

The average MCL estimate ranged between 74 and 83 dB HL in both groups and for both frequency ranges. The only appreciable difference between the two groups in terms of MCL was found at HFs and only in the right ear. The average slope of the loudness growth was slightly steeper for the HI listeners in the LF range (0.45 CU/dB for HI vs. 0.35 CU/dB for ONH) and substantially steeper in the HF range (0.8 CU/dB for HI vs. 0.45 CU/dB for NH). The average dynamic range was between 80 and 90 dB HL for the ONH listeners, and smaller for the HI listeners, especially at HFs (50.8 dB). Regarding the test–retest reliability, ACALOS showed an excellent reliability for estimating the hearing thresholds (ICC = 0.94; SEM = 4.5 dB), good reliability for estimating the MCL (ICC = 0.68, SEM = 6.5 dB), and very good reliability for estimating the slope (ICC = 0.82; SEM = 0.07 CU/dB). Overall, these results supported the inclusion of ACALOS in a clinical test battery, as it provides several outcomes (hearing thresholds, growth of loudness, MCL and dynamic range). ACALOS also showed a high time efficiency (around 10 min per ear).

## 6. Speech Perception in Quiet

### 6.1. Method

The word recognition score with four unforced choices (WRS-4UFC) test was proposed as a systematic and self-administered procedure that allows the estimation of supra-threshold deficits in speech perception in quiet. The speech material was the same as the one used for standard speech audiometry (Dantale I; Elberling et al., 1989) in Danish. The self-administered procedure consisted of the presentation of one word where the participant has to answer in a 4-unforced-choice paradigm (4UFC). After the acoustical presentation of each word, the target written word was assigned randomly to one of four buttons shown to the participant. The other three buttons contained words that were also taken from the Dantale-I corpus. They were chosen based on the lowest Levenshtein phonetic distance (Sanders and Chin, 2009) from the target. The term “unforced” corresponds to an additional choice, a question mark, that the listener can press if none of the four options are considered the right answer. Four lists of 25 words were presented at 40, 30, 20, and 10 dB above the individual PTA, in this order. A logistic function with two independent free asymptotes was fitted to the results from each individual ear and the speech reception threshold (SRTQ), at which 50% of the words were recognized, and maximum speech discrimination score (maxDS), which was the maximum value of the function (i.e., the upper asymptote). Both outcomes were estimated using psignifit 4 software (Schütt et al., 2016).

### 6.2. Results and Discussion

The results of the WRS-4UAFC outcome measures are shown in Table 6.

**Table 6:** Summary of the results of the word recognition scores (WRS-4UFC) test.

			**ONH**			**HI**		
**Outcome**	**Ear**		**Mean (SD)**	**Q1**	**Q3**	**Mean (SD)**	**Q1**	**Q3**
**measure**								
SRT_Q_ (dB HL)	LE		19.9 (7.1)	16.5	*19.2*	41.5 (13.5)	*31.8*	50.6
	RE		23.3 (8.9)	*17.2*	29.0	42.7 (12.6)	33.9	51.1
maxDS (%)	LE		99.2 (1.6)	100	100	97.2 (4.1)	*95.3*	100
	RE		97.2 (1.8)	95.5	97.6	93.9 (6.4)	92.1	98.4

*The results of the speech recognition threshold (SRT_Q_), and maximum discrimination score (maxDS) are shown as the mean, standard deviation, first, and third quantiles (25 and 75th percentiles) for each ear*.

The HI listeners' SRTQ were, on average, 20 dB higher than the ones of the ONH group. The interquartile range for the HI group was about 19 dB, whereas for the ONH group it was 3 dB for the left ear (LE) and 11.8 dB for the right ear (RE). The maxDS for both groups was close to 100%. However, the HI listeners showed larger variability, especially in the right ear (SD= 6.42%). In the analysis of the test–retest variability, the WRS-4UFC test showed poor to moderate reliability, especially at low levels (PTA + 10 dB; ICC = 0.25). However, at the higher presentation levels (i.e., individual PTA + 40 dB) the standard error of the measurement was only 4% (1 word). Regarding clinical applicability, the WRS-4UFC needs to be compared to traditional speech audiometry to explore the influence of using closed- vs. open-set and forced- vs. unforced-choice test procedures on the results.

## 7. Speech Perception in Noise

The Hearing in Noise Test (HINT; Nilsson et al., 1994) is an adaptive sentence recognition test carried out with speech-shaped noise. The following assumptions are considered in HINT (based on Plomp, 1978): (1) Speech materials made up of meaningful sentences yield a steep psychometric function; (2) stationary noise with the same spectral shape as the average spectrum of the speech material makes the speech reception threshold in noise (SRTN) less dependent on the spectral characteristics of the speaker's voice. Furthermore, the signal-to-noise ratio (SNR) between the target and masker is better defined across the frequency range. (3) The SRTN is independent of the absolute noise level as long as the noise level is above the “internal noise” level. Therefore, it is recommended to present the noise at least 30 dB above the “internal noise.” The internal noise is defined as the sum of the SRT in quiet of the tested listener and the SRT in noise for NH listeners, for a given speech material.

### 7.1. Methods

The Danish HINT was used as in Nielsen and Dau (2011) to obtain the SRTN but in a monoaural presentation. Additionally, a 20-sentence list was presented at a fixed SNR of +4 dB and scored to obtain a sentence recognition score (SScore^+4dB^). The presentation level of the noise was set between 65 and 85 dB SPL to ensure that the noise was always presented 30 dB above the individual PTA. Each ear was tested individually. All participants were tested using the same list with the same ear. Since small differences across lists were found in Nielsen and Dau (2011), this was done to ensure that all the listeners were tested with an equally difficult list. However, for the test–retest reliability study, the list and ear presented were randomized, only using lists 6–10. The listeners did not report recalling sentences from the test.

### 7.2. Results and Discussion

The results of the HINT outcome measures are shown in Table 7.

**Table 7:** Summary of the results of the hearing in noise test (HINT).

			**ONH**			**HI**		
**Outcome**	**Ear**		**Mean (SD)**	**Q1**	**Q3**	**Mean (SD)**	**Q1**	**Q3**
**measure**								
**SRT*_*N*_ (dB SNR)*	LE		1.0 (0.7)	0.4	1.5	4.1 (3.4)	1.4	6.7
	RE		–0.5 (1.1)	–1.0	0.0	2.6 (3.8)	0.0	4.2
SScore^+4*dB*^ (%)	LE		85.0 (11.7)	85	90	60.0 (26.6)	40	85
	RE		91.0 (9.6)	90	95	*62.3 (24.0)*	48.7	80

*The results of the speech recognition threshold in noise (SRTN), and sentence recognition score at +4 dB SNR (SScore^+4dB^) are shown as the mean, standard deviation, first, and third quantiles (25 and 75th percentiles) for each ear*.

The SRTN for ONH listeners were, on average, 2 dB higher than the ones reported in Nielsen and Dau (2011). This bias was also observed in the YNH listeners. However, this might be explained by the fact that they used diotic presentation, which can lead to a 1.5 dB improvement as reported by Plomp and Mimpen (1979). The results also showed a lower SRTN (1.5 dB) and higher SScore^+4dB^ (4%) for the right ear in both groups of listeners. According to Nielsen and Dau (2011), there was a significant main effect of test list. Such differences are seen mainly for lists 1–4, which were the lists used here. Therefore, the observed interaural difference can be ascribed to a list effect. However, it might be ascribed to other factors as, for example, a right-ear advantage as the one observed in NH listeners with tinnitus (Tai and Husain, 2018). Unfortunately, the difference across lists was not taken into account in the experimental design so that we cannot conclude that the difference is only due to the list effect. The ICC values (SRTN: ICC = 0.61; SScore^+4dB^: ICC = 0.57) indicated only moderate reliability of the HINT. The SRTN showed an SEM = 1.02 dB, which is below the step size of the test (2 dB). The SScore^+4dB^ showed an SEM value of 7.94%, which corresponds to an error in one of the sentences. However, the reliability of the test can be improved by using an adaptive method as the one described in Wagener et al. (2003); Rønne et al. (2017) where the SRTN estimation was optimized using a combination of word scoring and a maximum likelihood procedure.

## 8. Spectro-Temporal Modulation Sensitivity

A speech signal can be decomposed into spectral and temporal modulations. While speech-in-noise perception assessment leads to some confounds due to the variety of speech corpora, noise maskers, and test procedures that can all affect the results, the assessment of the contrast sensitivity of simpler sounds might be of interest for characterizing a listener's spectro-temporal processing abilities. Bernstein et al. (2013) showed significant differences between NH and HI listeners for detecting STM in random noise. These differences corresponded to specific conditions that were also useful for the prediction of speech-in-noise performance in the same listeners. Lately, the assessment of STM sensitivity in these specific conditions gained an increasing interest due to its potential for predicting speech intelligibility (Bernstein et al., 2016; Zaar et al., 2020) and for assessing cochlear-implant candidacy (Choi et al., 2016). Here, STM sensitivity was assessed using a new test paradigm that may be more suitable for a clinical implementation than the previous psychoacoustic versions of the test. The test was performed in two conditions: an LF condition (similar to the one previously used in Bernstein et al., 2016) and an HF condition (Mehraei et al., 2014).

### 8.1. Methods

The stimuli were similar to those of Bernstein et al. (2016) and Mehraei et al. (2014), but a different presentation paradigm was employed. A sequence of four noises was presented in each trial. The first and third stimulus always contained unmodulated noise, whereas the second and fourth stimuli could be either modulated or unmodulated. The test was performed with a low-frequency (LF) 3-octaves wide stimulus centered at 800 Hz (sSTM8 and fSTM8), and a 1-octave wide stimulus centered at 4 kHz (sSTM4k and fSTM4k). The stimuli were presented at 75 dB sound pressure level (SPL). After the sequence was presented, the listener had to respond whether the four sounds were different (“yes”) or the same (“no”). Two procedures involving catch trials were evaluated. The first test the screening spectro-temporal sensitivity (sSTM), a test consisting of 10 stimuli modulated at 20log(m) = –3 dB modulation level (ML), where m is the modulation depth, and five unmodulated ones presented in random order. The two runs of the two conditions could be completed in approximately 4 min. The outcome measure was the listener's contrast sensitivity (d')^3^ in the task. The second test was the “fast” spectro-temporal sensitivity (fSTM), which tracked the 80% point of the psychometric function using a yes/no task and the single-interval adjusted matrix (SIAM; Kaernbach, 1990) paradigm. The first two reversals were discarded and the thresholds were the average of the last four reversals. A negative response increased the modulation by 4 times the step size and by 5 times when there was a “caught.” These parameters were chosen for maximizing the attainability of the test after a pilot investigation. For the sSTM test, the stimulus was presented diotically, whereas for the fSTM the test was presented in each ear individually in a monoaural presentation.

### 8.2. Results and Discussion

The results of the spectro-temporal modulation sensitivity tests outcomes are shown in Table 8.

**Table 8:** Summary of the results of the spectro-temporal modulation sensitivity tests.

			**ONH**			**HI**		
**Outcome**	**Freq**.	**Ear**	**Mean (SD)**	**Q1**	**Q3**	**Mean (SD)**	**Q1**	**Q3**
**measure**	**Range**							
sSTM -3dB (d')	LF	Bin	2.6 (0.6)	2.4	3	1.7 (1.3)	0.4	3
	HF		1.6 (0.8)	1.1	2.4	0.6 (1.1)	–0.3	1.4
fSTM (dB ML)	LF	LE	–7.7 (1.8)	–9	–7.6	–2.8 (2.1)	–3.5	–0.8
		RE	–5.1 (3.1)	–7.2	–1.6	–1.6 (1.3)	–2	–0.6
	HF	LE	–8.0 (2.0)	–8.6	–6.2	–2.6 (2.4)	–3.8	–0.6
		RE	–5.6 (3.6)	–8.6	–2.1	–1.9 (1.5)	–2	–1

*The results of the screening STM sensitivity test (sSTM), and the threshold of the fast STM sensitivity test (fSTM) are shown for the low-frequency stimulus (8, because it is centered at 800 Hz) and high-frequency (4 k, centered at 4 kHz) as the mean, standard deviation, first, and third quantiles (25 and 75th percentiles) for each ear*.

The screening STM test (sSTM) shows the sensitivity in terms of d', where the maximum value is d' = 3, (i.e., 10 modulated and 5 unmodulated stimuli correctly detected). In the hypothetical case when all the catch trials are detected, the lowest d' value can be –0.3. The ONH listeners showed a high sensitivity in the LF condition (d' = 2.6) and a somewhat lower sensitivity in the HF condition (d' = 1.63) corresponding to 65% correct responses. The HI listeners showed a higher variability and a lower sensitivity in the LF condition (≈ 70% correct) and substantially lower sensitivity in the HF condition (0–50% correct responses). The threshold-tracking procedure (fSTM) showed results between –9 and –1.6 dB ML in the ONH group, whereas the HI listeners showed thresholds between –3.50 and –0.6 dB ML in the LF condition. Although the results of the fSTM LF condition were consistent with Bernstein et al. (2016), the results in the HF condition showed higher thresholds than the ones in Mehraei et al. (2014). This can be ascribed to the higher presentation level used in Mehraei et al. (2014) than in the current test procedure. According to Mehraei et al. (2014) fSTM4k could be a good predictor of frequency selectivity but, in the present study, the majority of the listeners presented elevated thresholds or could not complete the test. Therefore, this condition was excluded for further analysis. The fSTM showed an excellent reliability (ICC = 0.91; SEM = 0.93 dB ML) in the LF condition. However, several HI listeners were not able to complete the procedure for the HF condition. Overall, the use of the SIAM tracking procedure allowed us to obtain accurate thresholds, although additional repetitions were required, especially in the HF condition. This might be because the psychometric function for detecting the stimulus can be shallower in this condition, or because the 100% detection could not be reached even in the fully modulated trials. Therefore, a Bayesian procedure being able to estimate the threshold and slope of the psychometric function, such as the Bayes Fisher information gain (Figure: Remus and Collins, 2008), might be more suitable for this type of test. Another reason explaining the inability of the listeners to perform the test can be ascribed to the stimulus. Zaar et al. (2018) used a longer stimulus (1s), a diotic presentation, and a hearing loss compensation that ensured the audibility of the stimulus in all its frequency range. In their study, all the listeners were able to perform the tests and their sensitivity thresholds were well below the maximum value.

## 9. Extended Audiometry in Noise

The extended audiometry in noise (eAUD) is a tone detection test intended to assess different aspects of auditory processing by means of a task similar to pure-tone audiometry. The tone is presented in the presence of noise and the listener has to indicate whether the tone was perceived or not. The aspects of auditory processing assessed here are (1) tone-in-noise detection and (2) spectral and temporal resolution.

### 9.1. Tone-in-Noise Detection

In pure-tone audiometry, a given patient has to detect the simple stimulus (e.g., sinusioids) in quiet aiming at estimating the hearing thresholds of the listener. A simple way to explore the supra-threshold performance is to perform a tone-in-noise detection test by presenting noise at supra-threshold levels and obtaining the masked thresholds. However, the characteristics of the noise such as bandwidth, level, or inherit modulations can affect the results. Moore et al. (2000) proposed a test paradigm using a special type of noise, which is able to provide the same masking in the entire frequency range, so the hearing thresholds of an NH listener would raise according to the level of the noise. This is the so-called threshold-equalizing noise (TEN). The advantage of the TEN test is that the expected masked thresholds are similar to the level of the noise (i.e., as TEN is played at 70 dB per equal rectangular bandwidth (ERB), the masked threshold is expected to be at 70 dB SPL). Although this test was originally design to detect dead cochlear regions, recent evidence suggests that tone-in-noise detection can be representative of supra-threshold deficits beyond the audiogram (Schädler et al., 2020).

### 9.2. Spectro-Temporal Resolution

**Figure 3:**
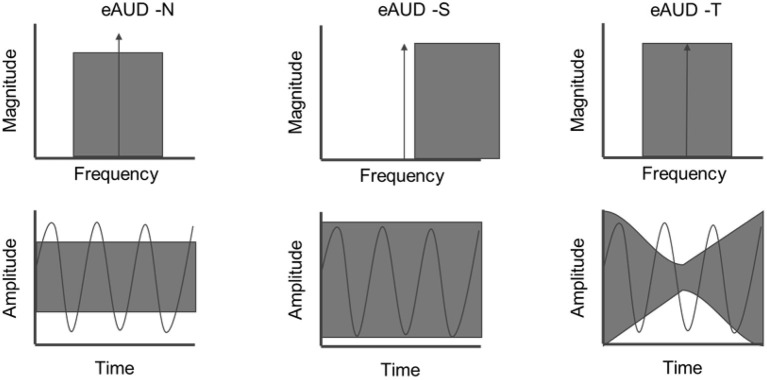
Sketch of the conditions of the spectro-temporal resolution measures of the extended audiometry in noise (eAUD). The top panel shows the spectrum of the noise and target pure-tone (delta), the bottom panel shows both signals in the time domain. Left panel: Tone in noise condition (eAUD-N). Middle panel: Spectral condition (eAUD-S). Right panel: Temporal condition (eAUD-T).

Frequency and temporal resolution are aspects of hearing that are fundamental for the analysis of perceived sounds. While NH listeners exhibit a frequency selectivity on the order of one-third of an octave when using isoinput levels (from Glasberg and Moore, 1990; Eustaquio-Martín and Lopez-Poveda, 2011), HI listeners typically have broader auditory filters, leading to impaired frequency selectivity (Moore, 2007). Temporal resolution can be characterized by the ability to “listen in the dips” when the background noise is fluctuating based on the so-called masking release (Festen and Plomp, 1990). Schorn and Zwicker (1990) proposed an elaborated technique for assessing both spectral and temporal resolution using two tests: (1) Psychoacoustical tuning curves and (2) temporal resolution curves. In both cases, the task consists of detecting a pure tone that is masked by noise or another tone while the spectral or temporal characteristics of the masker are varied. Later, Larsby and Arlinger (1998) proposed a similar paradigm, the F-T test, which was successfully tested in HI listeners (van Esch and Dreschler, 2011). However, the F-T test is based on a Bekesy-tracking procedure, which can be demanding and less reliable for some listeners than an adaptive procedure (Rhebergen et al., 2015). Here, the spectro-temporal resolution was assessed using a new test. This test is a tone-in-noise detection task consisting of three conditions as sketched in Figure 3.

1. eAUD-N: The tone is embedded in a 1-octave-wide threshold equalizing noise (TEN-HL; Moore, 2001). Because of the properties of the TEN-HL, the tone detection threshold is comparable to the level of the noise in dB HL.
2. eAUD-S: The tone is embedded in a TEN that has been shifted up in frequency. In the spectral domain, this yields spectral unmasking of the tone, so the detection threshold is lower than in eAUD-N.
3. eAUD-T: The tone is embedded in a temporally modulated noise with the same spectral properties as the one in eAUD-N. In the temporal domain, the modulations of the noise yield temporal unmasking, so the tone can be detected in the dips.

The outcome measures were focused on the temporal and spectral benefits expected in the eAUD-S and eAUD-T conditions compared to the eAUD-N condition. While in the noise condition (eAUD-N) the threshold is expected to be approximately at the level of the noise, in the temporal and spectral conditions the thresholds should be obtained at a lower level, showing temporal masking release (TMR) and spectral masking release (SMR).

### 9.3. Methods

The procedure used here was a yes/no task using a SIAM procedure (Kaernbach, 1990) similar to the one used in the eAUD-HF. Here, a TEN was presented together with a warble tone. If the target was detected, the target-presentation level is decreased according to a given step size; if it was not detected, the level is increased. If the stimulus was not presented (catch trial) but the listener provided a positive response, the level is decreased compared to the previous trial. As in the eAUD-HF, for each run, the threshold of each trial was calculated as the average of the last four reversals. The noise was presented at 70 dB HL. The LF condition corresponded to the detection of a 0.5 kHz warble tone, whereas the HF condition corresponded to a 2 kHz warble tone. The final threshold was calculated as the mean threshold of two repetitions. In the eAUD-S condition, the center frequency of the noise was *f*_*c*, noise_ = 1.1*f*_tone_. In the eAUD-T condition, the modulation frequency of the noise was set to, *f*_*m*_ = 4 Hz. The outcome measures of the eAUD are 1) the tone-in-noise threshold (TiN), (2) the temporal masking release (TMR), and (3) the spectral masking release (SMR). The first two reversals were discarded and the thresholds were the average of the last four‘reversals.

### 9.4. Results and Discussion

The results of the extended audiometry in noise outcomes are shown in Table 9.

**Table 9:** Summary of the results of the extended audiometry in noise (eAUD).

			**ONH**			**HI**		
**Outcome**	**Freq**.	**Ear**	**Mean (SD)**	**Q1**	**Q3**	**Mean (SD)**	**Q1**	**Q3**
**measure**	**Range**							
TiN (dB HL)	LF	LE	70.4 (4.5)	68	71.5	71.8 (2.6)	70.2	73.2
eAUD-N		RE	69.2 (4.6)	65.2	72.5	72.0 (2.8)	69.6	74.3
	HF	LE	71.1 (2.5)	69.7	72.7	74.7 (3.4)	72.5	76.1
		RE	70.8 (3.6)	70.5	71.7	74.2 (3.1)	72	76.2
TMR (dB)	LF	LE	7.5 (3.4)	6	7.5	7.7 (4.0)	6.1	10.1
eAUD (N -T)		RE	5.2 (3.3)	4	7.6	8.3 (2.7)	6.5	10.3
	HF	LE	13.0 (0.6)	12.7	13.2	7.9 (5.0)	5	11.6
		RE	10.7 (3.1)	9.1	10.2	8.1 (5.2)	5.1	10.7
SMR (dB)	LF	LE	19.3 (3.6)	16.5	21.7	19.6 (17.7)	17.7	23.2
eAUD (N -S)		RE	18.8 (4.6)	17	21.2	20.0 (5.2)	16.5	23.8
	HF	LE	26.8 (4.5)	27.5	29	19.3 (9.5)	12.1	26.3
		RE	27.2 (3.7)	26.2	29.5	19.5 (9.9)	12	26.8

*The results of the tone-in-noise (TiN), temporal masking release (TMR), and spectral masking release (SMR) are shown for the low-frequency condition (LF; 500 Hz) and high-frequency condition (HF; 2 kHz) as the mean, standard deviation, first, and third quantiles (25 and 75th percentiles) for each ear*.

The TiN showed a larger variance for the ONH group (SD = 4.5 dB HL) at LFs. The detection thresholds were in line with previous work with thresholds close to the noise presentation level (70 dB HL) (Vinay et al., 2017). The TMR shown by the NH group was larger at HFs (10 dB) than at LFs (7 dB). The HI group showed, on average, similar TMR only at LFs. The SMR shown by the ONH listeners was 19 dB for LFs and 26 dB for HFs. In contrast, for the HI listeners, the SMR was 7 dB lower only in the HF condition. The reliability of the eAUD was moderate for most of the conditions (ICC ≤ 0.75). The eAUD-S at LFs showed good reliability (ICC = 0.85; SEM = 1.78 dB). The masking release estimates showed good reliability only for the HF condition. The reason for this might be that masking release is a differential measure, and the cumulative error is, therefore, higher than that of each individual measure. The reduced reliability can be explained to some extent by the method used. To have a similar procedure as in pure-tone audiometry, the parameters of the SIAM tracking procedure were set accordingly. However, this made the test challenging and the listeners consistently missed several catch trials. Thus, extra trials were required to improve measurement accuracy, especially in the eAUD-N condition. Furthermore, the standard error of the measurement was in most cases larger than the final step size (2 dB). As in the case of the fSTM, a different procedure, such as Bayesian adaptive methods, might increase measurement reliability.

## 10. Binaural Processing Abilities

Binaural hearing is useful for sound localization and the segregation of complex sounds (Darwin, 1997). Interaural differences in level or timing are processed for spatial hearing purposes in the auditory system. In the case of hearing loss, the neural signal at the output of the cochlea can be degraded, which may lead to reduced binaural abilities typically connected to temporal fine structure (TFS) processing. Based on a method of estimating the upper-frequency limit for detecting an interaural phase difference (IPD) of 180° (IPDfmax Ross et al., 2007; Neher et al., 2011; Santurette and Dau, 2012), Füllgrabe and Moore (2017) recently proposed a refined test as a feasible way to evaluate TFS sensitivity. This paradigm was used in recent research that suggested that IPDfmax might be related to non-auditory factors (Strelcyk et al., 2019) and affected by factors beyond hearing loss, such as musical training (Bianchi et al., 2019). Therefore, the IPDfmax might be a task that requires auditory and non-auditory processing abilities beyond TFS sensitivity.

In contrast, binaural pitch detection assesses binaural processing abilities in a different manner. This test requires the detection of pitch contours embedded in noise, which are diotically or dichotically evoked. While the diotic condition can be resolved monoaurally, the dichotic condition requires the binaural processing abilities to be sufficiently intact to detect the contour. Previous studies showed that some listeners were unable to detect binaural pitch, regardless of the audiometric configuration (Santurette and Dau, 2012; Sanchez-Lopez et al., 2018). Therefore, it was of interest to compare the results of these two binaural processing tests.

Another approach for evaluating the binaural processing abilities is assessing binaural masking release (Durlach, 1963), which has been used in several studies (e.g., Strelcyk and Dau, 2009; Neher, 2017) and implemented in some commercial audiometers (Brown and Musiek, 2013). In this paradigm, a tone-in-noise stimulus is presented in two conditions: (1) a diotic condition where the tone is in phase in the two ears, and (2) a dichotic condition where the tone is in antiphase in the two ears. The difference between the two yields the benefit for tone detection due to binaural processing, the so-called binaural masking release (BMR).

### 10.1. Methods

The maximum frequency for detecting an IPD of 180° with pure-tones was obtained using a 2-AFC tracking procedure similar to the one used in Füllgrabe and Moore (2017). The stimuli were presented bilaterally in both ears as two sequencies of four tones. One sequence contained an ABAB sequence, where A means a diotic presentation and B an IPD of 180° between the tones presented to each ear, and the other an AAAA sequence. A positive response (detection) increased the frequency of the tone, and a negative response a decrease of the frequency. Although the stimuli duration and procedure were similar, the step size used here was slightly different, starting with steps of 2/3 octave and decreasing to a final step size of 1/6 octave in each reversal. The last six reversals were used for estimating the threshold. The frequency threshold (IPDfmax) was obtained from the average of two runs.

Binaural pitch detection scores were obtained using a clinical implementation of the test proposed by Santurette and Dau (2012). A 3-min sequence of noise was presented bilaterally. Ten diotic and ten dichotic pitch contours, embedded in the noise, had to be detected by the listener. The tones forming the pitch contours were generated by adding frequency-specific IPDs to the presented noise (Cramer and Huggins, 1958). The outcome measure of the binaural pitch test was the percentage score of detecting the dichotic pitch contours only, averaged across two repetitions (BP20).

The BMR was assessed using the same method as the extended audiometry. Two measurements were required: (1) tone-in-noise detection presented diotically (S_0_N_0_) and tone-in-noise detection presented dichotically, i.e., with the tone in anti-phase across the two ears (S_π_N_0_).

### 10.2. Results and Discussion

The results of the tests assessing binaural processing abilities are shown in Table 10.

**Table 10:** Summary of the results of the binaural processing abilities tests.

			**ONH**			**HI**		
**Outcome**	**Ear**		**Mean (SD)**	**Q1**	***Q3***	**Mean (SD)**	**Q1**	**Q3**
**measure**								
IPD_fmax_ (kHz)	Bin		0.76 (0.26)	0.59	0.98	0.69 (0.27)	0.52	0.88
BP_20_ (%)	Bin		87.5 (25.0)	87.5	100	80.7 (30.9)	70	100
BMR (dB)	Bin		16.5 (4.7)	13.5	17.5	14.7 (4.6)	12.2	*17.5*
(*S*_0_*N*_0_ – *S*_π_*N*_0_)								

*The results of the maximum frequency for IPD detection (IPD_fmax_), binaural pitch detection scores (BP_20_), and the binaural masking release (BMR) are shown as the mean, standard deviation, and first and third quantiles (25 and 75th percentiles) for each ear*.

The listeners in the ONH and HI groups showed IPDfmax thresholds around 700 Hz with a standard deviation (≈ 270 Hz) and interquartile range (≈ 370 Hz) similarly in both groups. These results are in line with the ones reported in Füllgrabe and Moore (2017). The IPDfmax test showed excellent reliability (ICC = 0.95; SEM = 65.4 Hz), and the median time needed for two repetitions was 10 min. This suggests that IPDfmax is a reliable measure of binaural processing abilities that can reveal substantial variability among both NH and HI listeners, which is valuable for highlighting individual differences among patients.

The overall results from the binaural pitch test for the NH listeners showed >87.5% correct detection, whereas the HI listeners' results showed a higher variability, with an interquartile range from 70 to 100%. The test showed excellent reliability (ICC = 0.98; SEM = 4%), which may be influenced by ceiling effects since 6 participants got 100% correct responses. Listeners reported a positive experience due to the test being short and easy to understand.

The BMR shown by both groups was around 15 dB, as expected from previous studies (Durlach, 1963). This BMR is, in essence, similar to the binaural masking level differences that are available in some clinical devices. However, in this test battery, the use of threshold equalizing noise provoked the S_0_N_0_ condition thresholds to be similar to the noise presentation level (i.e., 70 dB HL). The variability of this condition was substantially lower than the S_π_N_0_, suggesting that the use of S_π_N_0_ can be sufficient and more informative. A similar reasoning has been recently reported in Grant et al. (2021), where the dichotic condition alone was a sensitive auditory measure associated with the effects of noise exposure.

## 11. Exploratory Analysis

The collection of tests included in the test battery was intended to explore different and potentially independent aspects of hearing to obtain an auditory profile with controlled interrelations among the tests. A factor analysis performed in the HEARCOM study (Vlaming et al., 2011) based on data from 72 HI subjects revealed auditory dimensions: (1) HF processing, (2) audibility, (3) LF processing, and (4) recruitment. In the current study, the results of the behavioral tests were analyzed further in order to explore possible interrelations between the various outcome measures.

### 11.1. Methods

First, the data were pre-processed as in Sanchez-Lopez et al. (2018) to reduce the number of variables. The outcome variables of the frequency-specific tests were divided into LF (≤ 1 kHz) and HF (> 1 kHz) variables. This decision was supported by a correlation analysis performed on the complete set of outcome variables, where the outcomes corresponding to 2, 4, and 6 kHz as well as the ones corresponding to 0.25, 0.5, and 1 kHz were highly intercorrelated. For the tests performed monaurally, the mean of the two ears was taken as the resulting outcome variable. The resulting dataset (BEAR3 dataset^4^) contained 26 variables, divided into six groups corresponding to the six aspects of auditory processing considered here. The exploratory analysis consisted of a correlation analysis using Spearman correlations and factor analysis. The factor analysis was performed using an orthogonal rotation (“varimax”) and the method of maximum likelihood. The number of components was chosen to use parallel analysis, the resulting number of components was four.

### 11.2. Results

**Figure 4:**
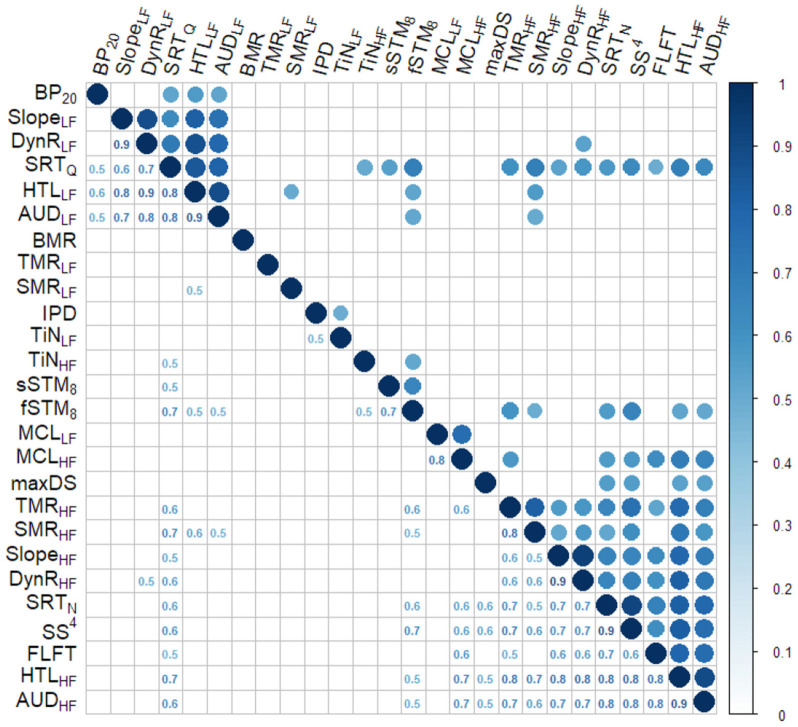
Correlation plot of the data set BEAR3. The upper part shows the significantly correlated variables as colored circles. The lower panel shows the numeric correlation value.

Figure 4 shows the results from the correlation analysis performed on the BEAR3 dataset. For convenience, the absolute value of the correlation was used when visualizing the data to show the strength of the correlation. The circles on the right-hand side of the figure depict significant correlations (*p* < 0.00001), and the correlation values are presented on the left-hand side of the figure. Two groups of correlated variables can be observed. The upper-left corner shows variables related to LF processing (dynamic range, the slope of the loudness function, and hearing thresholds) and speech intelligibility in quiet. The bottom-right corner shows a larger group of correlated variables including HF processing, speech intelligibility in noise, and spectro-temporal resolution at HFs. The variables that are not significantly interrelated are shown in the middle part of Figure 3, including the three variables related to binaural processing abilities (IPDfmax, BP20 and BMR), which were not significantly correlated to each other. The speech reception threshold in quiet (SRTQ) and the STM detection were correlated to various variables such as tone-in-noise detection, HF spectro-temporal resolution, LF hearing thresholds, and speech-in-noise perception.

The four factors resulting from the factor analysis showed 63% of explained cumulative variance. The variables with higher loadings (> 0.65) for each of the factors are shown in Table 11. The first factor, in terms of the amount of variance explained (19%), was associated with LF loudness perception and speech intelligibility in quiet, whereas the second factor (18% of variance explained) was associated with HF loudness perception. Despite loudness perception being associated with the first and second factor, the MCL was associated, both at HF and LF, with the third factor, while the fourth factor was associated with speech intelligibility in noise.

**Table 11:** Variables correlated to the four latent orthogonal factors resulting from the factor analysis with the method of maximum likelihood (ML).

	**ML2 (19%)**	**ML1 (18%)**	**ML3 (14%)**	**ML4 (12%)**
HTL_LF_	0.93			
DynR_LF_	–0.9			
AUD_LF_	0.82			
Slope_LF_	0.81			
SRT_Q_	0.67			
DynR_HF_		–0.93		
Slope_HF_		0.82		
HTL_HF_		0.79		
AUD_HF_		0.73		
MCL_HF_			0.92	
MCL_LF_			0.85	
SRT_N_				0.77
SScore^+4dB^				–0.78

*Columns are sorted in terms of the variance explained by each factor*.

## 12. General Discussion

The first goal of the present study was to collect data from a heterogeneous population of HI listeners, reflecting their hearing abilities in different aspects of auditory processing. The current study was motivated by the need for a new dataset to refine the data-driven approach for auditory profiling. The dataset should contain a representative population of listeners and outcome measures (Sanchez-Lopez et al., 2018) to allow a refined definition of the two types of auditory distortions and to identify subgroups of listeners with clinical relevance. To refine the data-driven auditory profiling, the BEAR3 dataset fulfills all the requirements discussed in Sanchez-Lopez et al. (2018). Other datasets containing a large number of listeners (e.g., Rönnberg et al., 2016; Gieseler et al., 2017) or physiological measures (e.g., Kamerer et al., 2019) could also be interesting for complementing the auditory profiling beyond auditory perceptual measures.

### 12.1. Relationships Across Different Aspects of Auditory Processing

The proposed test battery considers outcomes divided into six dimensions of auditory processing. One of the objectives of the study was to investigate the interrelations of different dimensions and measures. The present analysis showed two interesting findings. First, the correlation analysis showed two clusters of variables related to either LF or HF audiometric thresholds. Speech-in-noise perception was associated with HF sensitivity loss, temporal, and spectral masking release, whereas speech-in-quiet was correlated with both LF and HF hearing loss. Several outcomes were not interrelated, especially the outcomes associated with binaural processing abilities. Second, factor analysis yielded latent factors related to LF and HF processing, most comfortable level and speech in noise. Vlaming et al. (2011) showed four dimensions in the factor analysis of the HEARCOM project data corresponding to HF and LF spectro-temporal processing, MCL, and recruitment. In contrast, the current study showed that the slopes of the loudness growth, both at LF and HF, were not interrelated and contributed to the first and second latent factors. Additionally, the speech-in-noise test performed in HEARCOM was associated with the LF processing, whereas, in the present study, speech-in-noise dominates the fourth factor and is significantly correlated with HFs. The reason for this discrepancy might be the use of different types of noise (fluctuating masker) and test procedures in the two studies. Furthermore, in the HEARCOM study, the group participants included some younger hearing-impaired listeners and also participants with asymmetric or mixed hearing losses.

Overall, the data of the present study seem to be dominated by the audiometric profiles, with LF and HF processing reflecting the main sources of variability in the data. However, binaural processing abilities, loudness perception, and speech-in-noise outcomes showed a greater contribution to the variability of the supra-threshold measures than spectro-temporal processing outcomes.

### 12.2. Effects of the Participant's Cognitive Abilities

In this study, only auditory tests were considered. Indeed, some of the tests were quite demanding, which might have affected the results of listeners with reduced cognitive abilities. Here, only the age could be indicative of a likely cognitive decline, but it cannot confirm or deny this. In terms of age, we observed a significant effect on the results of the IPD and tone-in-noise tests. However, a thorough analysis on the effect of age was not carried out with the existing data. A more interesting approach would be to replicate this study including cognitive tests assessing executive functions, working memory or attention span with the aim of including a heterogeneous group of listeners with various cognitive abilities. Such a study could potentially assess both hearing and cognitive abilities toward a feasible test battery that can be included in the audiological assessment, either in or out of the clinic.

### 12.3. Extending the Test Battery to Other Clinical Populations

The proposed test battery was tested in a population of hearing-aid user candidates with various hearing abilities. However, the inclusion criteria left out the hearing-impaired listeners that were not likely to suffer from nonsyndromic presbycusis or noise-induced hearing loss. This means that adults with asymmetric hearing losses, severe-to-profound hearing loss, younger people with hearing deficits result of ototoxicity, or genetic conditions could potentially be included in a future study. Nevertheless, some tests might be affected by audibility or other aspects that have to be taken into account first. Furthermore, the test battery could be adapted to the pediatric clinical population, although that may be challenging for some tests.

### 12.4. Toward Clinical Feasibility of the Tests

The test–retest reliability of the test battery was investigated based on the results of a subset of listeners who participated 2–5 months after the first visit. The analysis was based on the ICC and the SEM. Some of the tests, such as IPDfmax, binaural pitch, and eAUD-HF (FLFT), showed good to excellent test–retest reliability with all ICC values above 0.9, while other tests, such as the extended audiometry in noise and speech intelligibility in quiet, showed poor reliability. The selected tests were conducted in two sessions and the total time was, on average, 3 h including the instructions and interview. In realistic clinical setups, a subset of tests with high reliability and a reasonably low difficulty would need to be prioritized. For a clinical version of the test battery, other tracking procedures such as Bayesian functional information (Remus and Collins, 2008) might be adopted to improve the reliability and time efficiency in some tasks such as STM and tone detection in noise. Moreover, if time-efficiency is crucial, testing some aspects of auditory processing out of the clinic, as other proposed test batteries for auditory research (Lelo De Larrea-Mancera et al., 2020), might be a solution for completing the patient's hearing profile. The use of speech-in-noise tests can be a useful tool for the characterization of the listener's hearing deficits that can be performed under different conditions, including monaural, binaural, unaided, and aided stimuli presentations. While here the tests were performed monaurally and unaided, a binaural condition as well as at least one aided measure (i.e., with hearing aids) could also be included in clinical practice. A clinical test battery with the subset of tests that showed a good or excellent test–retest reliability should be evaluated in a large scale study. In this study, we explored the use of an extended audiometry using the same test procedure for assessing high-frequency audibility (eAUD-HF), tone-in-noise detection (eAUD-N), spectro-temporal resolution (eAUD-S and eAUD-T), and binaural processing abilities. This procedure can be further explored and be performed by a hearing-care professional rather than in the current experimental setup. However, if the goal is to accurately estimate the hearing deficits of the patient, the test battery should include several aspects of auditory processing and provide detailed information on the supra-threshold deficits of the patient. The tests that showed potential for the clinical implementation were ACALOS, HINT, fSTM (only the LF condition), Binaural Pitch, and IPD_fmax_. Such a test battery could serve to identify a clinically relevant subset of patients (auditory profiles) that may benefit from specific types of hearing rehabilitation toward a “stratified approach” (Trusheim et al., 2007) for audiology practice.

### 12.5. Toward Personalized Rehabilitation Based on Hearing Deficits

The present study was motivated by a novel approach for hearing loss characterization, recently proposed in Sanchez-Lopez et al. (2018). In their study, a data-driven profiling method was able to identify four relevant groups of listeners with a large similarity within each group and a substantial dissimilarity across groups. This stratification was possible by using two abstract dimensions (distortion type-I and distortion type-II) that can characterize each individual's hearing deficits. The dataset obtained in the present study was analyzed using the same approach in Sanchez Lopez et al. (2020) showing that speech intelligibility-related deficits and loudness-related deficits were associated with the two abstract and orthogonal dimensions. Moreover, four relevant subpopulations were proposed as the auditory profiles. Although the proposed data-driven method is constrained and other relevant subpopulations may be found using a less restrictive approach, the current definition of the four auditory profiles allows a necessary simplification of the variety of hearing impairments than enables a meaningful stratification.

Following the principles of stratified medicine (Trusheim et al., 2007), the first criterion for implementing personalized treatments is that the identification of the patient subpopulations must be technically feasible. In stratified medicine, the patient's phenotype is usually obtained by clinical biomarkers, which are measurable characteristics that associate the optimal treatment to a patient subpopulation. In the present study, different perceptual measurements are proposed as candidates for establishing the association between the heterogeneous hearing deficits and potential target treatments. However, before the clinical biomarkers can be established, patient subpopulations with a likely different response to different treatments must be identified. This has been explored in Wu et al. (2020, 2021) and Sanchez Lopez et al. (2021). A profile-based hearing-aid fitting process has also been implemented in a large field study within the BEAR project. In that study, the patients will be tested with a subset of the tests presented here, fitted with hearing aids, and their aided performance and self-perceived benefit were evaluated in and out of the clinic. This is expected to set the basis for targeted interventions involving not only hearing-aid fitting, but also the use of new tools for evaluating the experiences of hearing-aid users (Lund et al., 2020) and recommendations for individualized pathways (Sanchez-Lopez et al., 2021a).

## 13. Conclusion

The current study has shown the rationale behind the BEAR test battery and the selected tests for characterizing hearing deficits in listeners with various hearing abilities. The analysis of the data showed that a reduced BEAR test battery has potential for clinical implementation, providing relevant and reliable information reflecting several auditory domains. The proposed test battery showed good reliability, was reasonably time-efficient, and easy to perform. The implementation of a clinical version of the test battery is to be evaluated in future research, e.g., in a larger field study to further refine the auditory profiling approach. Moreover, the current data have been already analyzed for the purpose of auditory profiling (Sanchez Lopez et al., 2020), showing the potential of this test battery for hearing rehabilitation.

## Data Availability Statement

The datasets presented in this study can be found in online repositories. The names of the repository/repositories and accession number(s) can be found below: https://doi.org/10.5281/zenodo.3459579.

## Ethics Statement

The studies involving human participants were reviewed and approved by Science-Ethics Committee for the Capital Region of Denmark H-16036391. The patients/participants provided their written informed consent to participate in this study.

## Author Contributions

Author contributions according to CRediT (Contributor Roles Taxonomy). RS-L: conceptualization, methodology, software, validation, formal analysis, investigation, data curation, visualization, and writing-original draft and editing. SN: investigation, validation, resources, formal analysis, visualization, and writing-original draft. ME-H-A: methodology, investigation, resources, and writing-review. MF and FB: conceptualization, methodology, supervision, and writing-editing. MW and OC: investigation, resources, and writing-review. TN: methodology, supervision, project administration, and writing-review. SS and TD: conceptualization, methodology, supervision, project administration, funding acquisition, and writing-review. All authors contributed to the article and approved the submitted version.

## Funding

This work was supported by Innovation Fund Denmark Grand Solutions 5164-00011B (Better hEAring Rehabilitation project), Oticon, GN Resound, Widex, and other partners (Aalborg University, University of Southern Denmark, the Technical University of Denmark, Force Technology, Aalborg, Odense, and Copenhagen University Hospitals).

## Conflict of Interest

RS-L was employed by the company Interacoustics A/S, Denmark. FB was employed by the company Oticon Medical, Denmark. SB was employed by company Oticon A/S. The remaining authors declare that the research was conducted in the absence of any commercial or financial relationships that could be construed as a potential conflict of interest.

## Publisher's Note

All claims expressed in this article are solely those of the authors and do not necessarily represent those of their affiliated organizations, or those of the publisher, the editors and the reviewers. Any product that may be evaluated in this article, or claim that may be made by its manufacturer, is not guaranteed or endorsed by the publisher.

## Footnotes

^1^The factors identified correspond to the authors' conclusions based on cited references. For example, Johannesen et al. (2016) identified the basilar membrane compession as a predictor of speech intelligibility in stationary noise and temporal processing as a predictor of speech-in-speech intelligibility. Rhebergen et al. (2006), Oxenham and Simonson (2009), and Desloge et al. (2017) identified the audibility of the soft speech sounds in the presence of fluctuating maskers as a crucial factor for speech intelligibility.

^2^While other listeners presented PTA ≤ 22 dB, the individual thresholds did not fulfill this criteria.

^3^d' was defined as $Z\left(\frac{N_{H}+0.5}{H+1}\right)-Z\left(\frac{N_{FA}+0.5}{FA+1}\right)$, where Z refers to the z-score transformation, H is the total number of target presentations, and FA is the total number of catch trials.

^4^The BEAR3 available at Zenodo contains an observation labeled “0,” which corresponds to the results of one of the examiners and it is not used in the present analysis.
