# Corrigendum: Auditory tests for characterizing hearing deficits in listeners with various hearing abilities: The BEAR test battery

**DOI:** 10.3389/fnins.2022.1122830

**Published:** 2023-01-25

**Authors:** Raul Sanchez-Lopez, Silje Grini Nielsen, Mouhamad El-Haj-Ali, Federica Bianchi, Michal Fereczkowski, Oscar M. Cañete, Mengfan Wu, Tobias Neher, Torsten Dau, Sébastien Santurette

**Affiliations:** ^1^Hearing Systems Section, Department of Health Technology, Technical University of Denmark, Kgs. Lyngby, Denmark; ^2^Interacoustics Research Unit, Kgs. Lyngby, Denmark; ^3^Institute of Clinical Research, University of Southern Denmark, Odense, Denmark; ^4^Oticon Medical, Smørum, Denmark; ^5^Research Unit for ORL-Head & Neck Surgery and Audiology, Odense University Hospital & University of Southern Denmark, Odense, Denmark; ^6^Centre for Applied Audiology Research, Oticon A/S, Smørum, Denmark

**Keywords:** audiology, hearing loss, loudness, binaural processing, speech perception, spectro-temporal resolution, auditory profile

In the published article, there was an error in **Equation (1)** as published. **The right part of the equation contained a sinusoid with** ***f*** = ***f***_***c***_** (stimulus frequency), where the correct formulation is with** ***f*** = ***f***_***r***_ **(frequency rate)**. The corrected **Equation (1)** appear below.


(1) $$\begin{array}{l} wt\left(f_{c},t\right)=\sin\left(2\pi f_{c}t+\frac{f_{c}f_{e}}{f_{r}}\sin\left(2\pi f_{r}t\right)\right), \end{array}$$

Additionally, the email address of the corresponding author RS-L has been updated from rsalo@dtu.dk to raul.sanchezlopez@igdore.org.

The authors apologize for this error and state that this does not change the scientific conclusions of the article in any way. The original article has been updated.

