# Supplementary material for: Auditory Tests for Characterizing Hearing Deficits in Listeners With Various Hearing Abilities: The BEAR Test Battery
Source: Front Neurosci. 2021 Sep 29;15:724007. doi: 10.3389/fnins.2021.724007 (PMC8512168; doi:10.3389/fnins.2021.724007)
Supplement: Supplementary file 1 [file Data_Sheet_1.pdf]

Supplementary Material of the article: "Auditory tests for characterizing hearing deficits in listeners with various hearing abilities: The BEAR test battery"

1 DETAILS OF THE TEST-RETEST RELIABILITY STUDY

Intraclass correlation (ICC) is a way of measuring the reliability of a measurement method (Stratford and Goldsmith, 1997). A higher ICC value (ranges between 0 - 1) indicates a higher correlation between the test and retest measurement, which is an indication of higher reliability. It is important to state that there is no standard value for acceptable reliability, and a low ICC could not only reflect the low degree of measurement agreement but also relate to the lack of variability among the sampled subjects. Koo and Li (2016) suggest that an ICC value of less than 0.5 is an indication of poor reliability. Values varying between 0.5 and 0.75 indicated a mediate reliability, and an ICC value of above 0.75 indicates good reliability. An ICC value of above 0.9 indicated excellent reliability of the measurement method. Both the Pearson's r and the ICC can give misleading results, as they are very sensitive to the spread of data between subjects. Therefore, Downham et al. (2005) suggest to also investigate if there is a systematic bias of the data and a measurement error. In this study, the test-retest reliability of the test battery has been explored by using the intraclass correlation (Koo and Li, 2016), Pearson's correlation, systematic change in means (Downham et al., 2005) and standard error of measurements (SEM; Stratford and Goldsmith, 1997).

1.1 Measures of reliability

Interclass cross-correlation calculation has different forms based on different assumptions. By looking at the flowchart presented in the paper by Koo and Li (2016), a two-way mixed effect model was chosen for the analysis. The absolute agreement was chosen, as Koo and Li (2016) stated that the test-retest reliability study would be meaningless if there was no agreement between repeated measures. Depending on the test, either a single measurement or the mean of k measurements was used as the type of measurement. The equation below shows the formula for the two-way mixed model, with absolute agreement

$$ICC = \frac{MR_R - MS_E}{MS_R + (k-1) + \frac{MS_E + k}{n(MS_C - MS_E)}} \tag{S1}$$

where MS_R = mean square for rows; MS_E = mean square for error; MS_C = mean square for columns; n = number of subjects and k = number of raters/measurements. Pearson's correlation (R) is another way of investigating the reliability and gives very similar results to the ICC. While the ICC is looking at the distance of the point from a straight line that is going through the origin, Pearson's r is looking at the distance from any kind of linear line (Koo and Li, 2016). For investigating if there is a systematic bias of the data and a measurement error, both the mean difference in results between the two sessions and the 95% confidence interval is calculated for all tests. If the mean difference ($\bar{d}$) had a negative value, this indicates that the results from the first session tend to be larger than the second one. If the confidence interval is including zero, it can be concluded that there is no systematic bias between the two sessions. The standard error of measurement (SEM) is also calculated. SEM is a way of calculating measurement error (Goldsmith Stratford, 1997) and is a way to compare different measurement methods. Because it is in the same units as the original measure, also the SEM is calculated in percentage to compare different measurement method

Table S1. The ICC and Pearson's R values for all tests of the test battery, together with the systematic difference in means and standard error of the measurements.

Test	Condition	ICC	R	Systematic change	SEM (%)
WRS	10dB	0.591, p = 0.001	0.59	d = 0.04, CI = [-0.04,0.11]	0.13 (23.05)
	20dB	0.291, p = 0.096	0.28	d = -0.007, CI = [-0.06,0.04]	0.078 (9.84)
	30dB	0.251, p = 0.128	0.25	d = -0.005, CI = [-0.02,0.01]	0.03 (3.16)
	40dB	0.475, p = 0.011	0.48	d = -0.009, CI = [-0.03, 0.01]	0.04 (4.29)
HINT	SRT_N	0.611, p = 0.001	0.60	d = 0.17, CI = [-0.46,0.79]	1.02 (211.54)
	SS^{+4dB}	0.574, p = 0.002	0.58	d = -2.96, CI = [-7.73,1.82]	7.94 (9.56)
STM	LF	0.916, p = 0.00	0.85	d = -0.09 CI = [-0.84, 0.67]	0.93 (12.26)
	HF	0.548, p = 0.003	0.59	d = 0.51, CI = [-0.27, 1.29]	1.31 (37.4)
ACALOS	HTL	0.946, p = 0.000	0.95	d = -0.13, CI = [-1.27, 1.00]	4.59 (17.53)
	MCL	0.678, p = 0.000	0.68	d = 0.53, CI = [-1.10, 2.16]	6.59 (7.86)
	Slope	0.821, p = 0.000	0.82	d = -0.002, CI = [-0.02, 0.02]	0.07 (15.51)
Binaural Pitch	Dichotic	0.987, p = 0.000	0.99	d = -2, CI = [-5.54, 1.54]	3.99 (4.91)
	Total	0.983, p =0.000	0.99	d = -0.5, CI = [-2.61, 1.61]	2.27 (2.52)
Frequency tracking procedures	IPD_{fmax}	0.950, p = 0.000	0.96	d = -15.84, CI = [-66.44, 34.75]	65.39 (6.37)
	FLFT	0.890, p = 0.000	0.89	d = 212.71, CI = [-89.7, 515.1]	495.3 ()
eAUD-B	S0N0	0.327, p = 0.101	0.41	d = 1.99, CI = [0.24, 3.74]	2.28 (3.24)
	$S\pi N0$	0.673, p = 0.007	0.70	d = 1.10, CI = [-1.62, 3.83]	3.1 (5.48)
	BMR	0.783, p = 0.002	0.77	d = 0.89, CI = [-1.08, 2.86]	2.25 (16.2)
eAUD-N	TiN_{LF}	0.325, p = 0.05	0.40	d = 1.27, CI = [0.09, 2.44]	2.02 (2.87)
	TiN_{HF}	0.551, p = 0.005	0.54	d = 0.29, CI = [-0.99, 1.56]	2.11 (2.89)
eAUD-S	S_{LF}	0.851, p = 0.00	0.85	d = -0.36, CI = [-1.45, 0.73]	1.78 (3.34)
	S_{HF}	0.954, p = 0.000	0.95	d = 0.51, CI = [-0.66, 1.69]	1.92 (4.08)
	SMR_{LF}	0.651, p = 0.004	0.68	d = 1.48, CI = [0.04, 2.91]	2.47 (14.24)
	SMR_{HF}	0.858, p = 0.000	0.85	d = -0.32, CI = [-2.09, 1.46]	2.85 (11.19)
eAUD-T	T_{LF}	0.665, p = 0.002	0.77	d = 1.35, CI = [0.49, 2.21]	1.64 (2.59)
	T_{HF}	0.875, p = 0.000	0.89	d = -0.96, CI = [-2.00, 0.09]	1.78 (2.88)
	TMR_{LF}	0.192, p = 0.205	0.19	d = -0.06, CI = [-1.40, 1.27]	2.17 (30.24)
	TMR_{HF}	0.668, p = 0.003	0.71	d = 1.13, CI = [-0.38, 2.63]	2.54 (23.96)

with different units.

$$SEM = \sigma_T \sqrt{(1 - ICC)}$$

(S2)

where σ_T is the total sample standard deviation, and ICC is the ICC value shown in equation 1.1.

$$SEM(\%) = \frac{SEM}{\bar{m}} 100 \qquad \text{(S3)}$$

where $\bar{m}$ is the mean of all measurements.

1.2 Results and discussion

Summary of the results are shown in Table S1.

The test-retest reliability of the test battery has been investigated, looking at the ICC, Pearson's R, systematic changes in the data and the SEM. Some tests, such as IPD, Binaural Pitch and FLFT showed a good to excellent test-retest reliability with all ICC values above 0.89. There was also no indication of a systematic bias, and the SEM showed low values that were below 7% of the total mean for each test. The ACALOS outcome measures also showed good reliability with ICC ranging from 0.67 to 0.95 (ICC(HTL) = 0.95, ICC(MCL) = 0.67, ICC(Slope) = 0.82). There was no indication of a systematic change in the data, and the SEM values for both, the HTL and MCL, varied around 5 dB, which was the same as the

uncertainty in the one expected in pure-tone audiometry. For the WRS test, lower ICC values were found, indicating poorer reliability. This could be a result of the participants having the alternatives visually in front of them, and even though they could not hear the word, they chose the word closest to it. The mean difference between test and retest for the 30 dB and 40 dB conditions is 4%, which is only one difference of incorrect words (1/25). A mediate reliability was shown for both outcomes of the HINT measurements, with ICC varying between 0.57 and 0.61. One reason for this mediate reliability could be the choice of lists and lack of randomization in the first session. As mentioned in the main document, there was a small list effect between the two ears that can also play a role here. There was no indication of any systematic changes in the data, and the SEM values were relatively small, which indicates good reliability. The spectro temporal modulation sensitivity (STM) measurements showed an excellent reliability for the LF condition (ICC($fSTM_8$) = 0.91) and a mediate reliability for the HF condition (ICC($fSTM_{4k}$) = 0.548). In addition, the SEM values showed better reliability for the LF condition. A reason for this could be that many subjects could not simply detect any modulation for the HF condition and therefore answered randomly. A poor to mediate reliability is shown for each condition of the binaural extended audiometry (eAUD-B; ICC(S0N0) = 0.327, ICC(Sπ N0) = 0.673, ICC(BMR) = 0.783). Diotic condition (S0N0) showed the lowest ICC value and the lowest spread of the data. However, there was also a shift in the data, with higher values for the first session. For the SpiN0 condition and the BMR, there was no shift in the data. The S0N0 was used for calculating the BMR, and reliability can be questionable. The TiN condition of the extended audiometry (eAUD-N) showed a poor to mediate reliability (ICC(TiN_{LF}) = 0.325, ICC(TiN_{HF} = 0.551). The results of the LF condition also showed a shift towards higher values for the first session. The TiN part of the extended audiometry was used for calculating both the SMR and the TMR which is crucial to understand the following results. The temporal condition showed mediate reliability, and a systematic change showing higher values for the first session. The spectral condition of the extended audiometry (eAUD-S) showed results that are promising for its implementation in the clinics with a good to excellent reliability for both conditions (ICC(S_{LF}) = 0.851, ICC(S_{LF}) = 0.954), however, the reliability of the spectral masking release was lower. Moreover, all conditions of the extended audiometry show somehow the same standard error of measurements (SEM), around 2 dB, which is the same as the minimum step size.

REFERENCES

Downham, D. Y., Holmbäck, A. M., and Lexell, J. (2005). Reliability of measurements in medical research and clinical practice. In *Studies in Multidisciplinarity Vol 3* (Elsevier), chap. 9. 147–163. doi:10.1016/S1571-0831(06)80013-4

Koo, T. K. and Li, M. Y. (2016). A guideline of selecting and reporting intraclass correlation coefficients for reliability research. *Journal of chiropractic medicine* doi:10.1016/j.jcm.2016.02.012

Stratford, P. W. and Goldsmith, C. H. (1997). Use of the standard error as a reliability index of interest : An applied example using elbow flexor strength data. *Physical Therapy* 77, 745–750. doi:10.1093/ptj/77.7.745
